# A four gene signature predicts benefit from anthracyclines: evidence from the BR9601 and MA.5 clinical trials

**DOI:** 10.18632/oncotarget.5562

**Published:** 2015-09-10

**Authors:** Melanie Spears, Fouad Yousif, Nicola Lyttle, Paul C. Boutros, Alison F. Munro, Chris Twelves, Kathleen I. Pritchard, Mark N. Levine, Lois Shepherd, John MS. Bartlett

**Affiliations:** ^1^ Transformative Pathology, Ontario Institute for Cancer Research, MaRS Centre, Toronto, ON, Canada; ^2^ Informatics and Bio-computing, Ontario Institute for Cancer Research, MaRS Centre, Toronto, ON, Canada; ^3^ Department of Medical Biophysics, University of Toronto, Toronto, ON, Canada; ^4^ Department of Pharmacology and Toxicology, University of Toronto, Toronto, ON, Canada; ^5^ Edinburgh Cancer Research UK Centre, MRC IGMM, University of Edinburgh, Crewe Road South, Edinburgh, UK; ^6^ Leeds Institute of Cancer and Pathology and Cancer Research UK Centre, St James’ University Hospital, Leeds, UK; ^7^ Sunnybrook Odette Cancer Centre, Toronto, ON, Canada; ^8^ University of Toronto, Toronto, ON, Canada; ^9^ McMaster University and Hamilton Health Sciences, Hamilton, ON, Canada; ^10^ NCIC Clinical Trials Group (NCIC CTG] and Queen's University, Kingston, ON, Canada

**Keywords:** breast cancer, anthracycline, chromosome instability, predictive biomarker

## Abstract

Chromosome instability (CIN) in solid tumours results in multiple numerical and structural chromosomal aberrations and is associated with poor prognosis in multiple tumour types. Recent evidence demonstrated CEP17 duplication, a CIN marker, is a predictive marker of anthracycline benefit. An analysis of the BR9601 and MA.5 clinical trials was performed to test the role of existing CIN gene expression signatures as predictive markers of anthracycline sensitivity in breast cancer.

Univariate analysis demonstrated, high CIN25 expression score was associated with improved distant relapse free survival (DRFS) (HR: 0.74, 95% CI 0.54-0.99, *p* = 0.046). High tumour CIN70 and CIN25 scores were associated with aggressive clinicopathological phenotype and increased sensitivity to anthracycline therapy compared to low CIN scores. However, in a prospectively planned multivariate analysis only pathological grade, nodal status and tumour size were significant predictors of outcome for CIN25/CIN70. A limited gene signature was generated, patients with low tumour CIN4 scores benefited from anthracycline treatment significantly more than those with high CIN4 scores (HR 0.37, 95% CI 0.20-0.56, *p* = 0.001). In multivariate analyses the treatment by marker interaction for CIN4/anthracyclines demonstrated hazard ratio of 0.35 (95% CI 0.15-0.80, *p* = 0.012) for DRFS. This data shows CIN4 is independent predictor of anthracycline benefit for DRFS in breast cancer.

## INTRODUCTION

Meta-analyses performed by the Early Breast Cancer Trialists Collaborative Group (EBCTCG) confirm a significant increase in disease free and overall survival (OS) following the addition of anthracyclines to polychemotherapy [1]. Anthracyclines, however, cause significant toxicities including life threatening cardiotoxicity and leukaemia [2–4]. Selecting the subset of patients who will benefit from adjuvant anthracycline whilst sparing other patients unnecessary toxicities remains a clinically highly relevant approach for early breast cancer. Significant resources have been devoted to developing markers that may predict anthracycline benefit (HER2, TOP2A, Ch17CEP and TIMP1) with limited success [5–9]. To date Ch17CEP (duplication of the peri-centromeric α-satellite region of chromosome 17) and TOP2A are the only markers that have shown consistent results across a number of clinical trials [7–9]. The functional pathways related to anthracycline benefit in Ch17CEP/TOP2A altered tumours remain unknown. Research from our group has linked the predictive effect of CEP17 *in vivo* to chromosome instability (CIN), which itself is predictive of anthracycline benefit in the BR9601 trial [10] and in preclinical models.

CIN is a phenotype description of genomic instability at the karyotypic level that results in multiple alterations in chromosomal number or structure. Multiple mechanisms drive CIN, including compromised spindle assembly checkpoint (SAC), sister chromatid cohesion defects, additional centrosomes and abnormal spindle kinetochore attachments. Pre-mitotic mechanisms may also include defects in DNA repair and replication pathways. CIN is associated with poor prognosis in many patients with solid tumours [11–12]. Critically for the current study, cell lines with high CIN phenotype enter mitotic catastrophe if challenged with anthracyclines, possibly as a result of defective SAC and other G2/M checkpoints [13]. *In silico* analysis has identified two mRNA signatures associated with CIN, “CIN25” and “CIN70”, as predictive of prognosis in a number of cancer datasets [14]. Furthermore high CIN70 signature expression was associated with paclitaxel resistance in ovarian cancers [15]. The CIN70 signature incorporates many genes whose mRNA expression levels correlate with proliferation, and have a role in the cell cycle [11].

Our work and that of others support a link between SAC dysregulation, a potential cause of CIN, CIN itself [10] and markers of CIN [7–9] and benefit from anthracycline containing polychemotherapy *in vivo*. Preclinical evidence linking CIN to anthracycline sensitivity support our hypothesis that CIN provides a potential clinically useful and relevant means of selecting those patients who are likely to benefit from anthracycline containing chemotherapy. Successful validation of such an approach would further support the selective use of anthracycline based chemotherapy and provide a viable diagnostic approach to support such selective use.

To further validate the role of existing CIN gene expression signatures as markers of anthracycline sensitivity we assessed these gene expression signatures in a prospectively planned and powered retrospective analysis of two pivotal clinical trials (BR9601 and MA.5). In addition we identified a new minimal gene set encapsulating the predictive value of these assay and validated its ability to stratify patients according to anthracycline benefit using clinical outcome.

## RESULTS

### Correlation of CIN25 and 70 and clinicopathological parameters with clinical outcomes

We successfully analysed 282 of 321 (87.9%) and 421 of 440 (95.7%) tumours from BR9601 and MA.5, respectively. High CIN25 and CIN70 scores were defined as above the median as previously described. In univariate analysis using continuous clinicopathological biomarkers, high CIN25 and CIN70 scores were associated with younger age (*p* < 0.0001), high tumour grade (*p* < 0.0001), PgR negativity (*p* < 0.0001) and ER negativity (*p* < 0.0001) but not with tumour size, nodal status or HER2 status.

### CIN signatures as prognostic markers for OS and DRFS

In a preplanned analysis the prognostic significance of CIN25 and CIN70 was tested on the entire patient cohort, irrespective of allocated adjuvant chemotherapy or trial. No significant association between CIN70 expression and DRFS (HR: 1.14, 95% CI 0.91-1.43, *p* = 0.273) or OS (HR: 1.14, 95% CI 0.88-1.45, *p* = 0.278) was evident. By contrast, tumours with high CIN25 scores were associated with reduced DRFS (HR: 1.43, 95%CI 1.11-1.67, *p* = 0.004, Figure 2A) and OS (HR: 1.45, 95%CI 1.14-1.85, *p* = 0.003, Figure 2B). After multivariate analysis and adjustment for nodal status, grade, size, age, HER2, ER and PgR status, a high CIN25 score was not an independent predictor for DRFS or OS.

**Figure 1:**
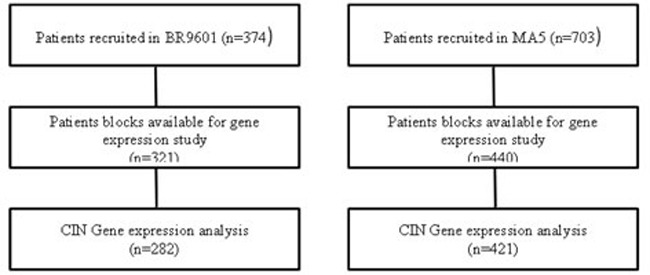
Schematic REMARK diagram representing the BR9601 and MA.5 cohorts

**Figure 2:**
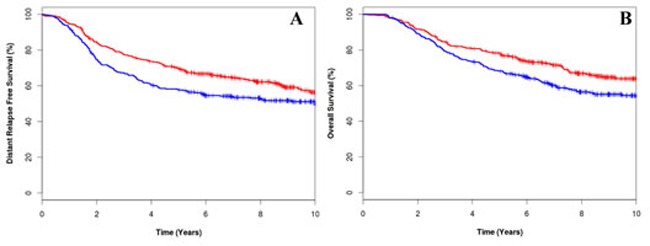
Kaplan-Meier survival curves for low CIN25 score [red line] and high CIN25 score (blue line) for distant relapse free survival (A) and overall survival (B)

### CIN signatures as predictive markers of anthracycline benefit

In a preplanned analysis no significant differential benefit in either DRFS or OS from E-CMF treatment was demonstrated in univariate analysis between patients whose tumours had high or low CIN70 expression (Table 1).

**Table 1:** Hazard ratios for overall survival and distant relapse free survival comparing epirubicin plus cyclophosphamide, methotrexate and fluorouracil [E-CMF] with CMF alone by biomarker status

	Distant Relapse Free Survival					
	Low Biomarker		High Biomarker		Treatment*Marker	
	HR	95% CI	HR	95% CI	HR	Test for Interaction *P*
CIN70	0.79	0.57-1.10	0.79	0.58-1.08	0.97	0.904
CIN25	0.85	0.61-1.21	0.74	0.54-0.99	0.86	0.519
CIN25 in grade III	0.81	0.51-1.30	0.66	0.46-0.94	0.81	0.479
CIN25 in grade I & II	0.85	0.50-1.43	1.12	0.58-2.12	1.30	0.541
	**Overall Survival**					
	Low Biomarker		High Biomarker		Treatment*Marker	
	HR	95% CI	HR	95% CI	HR	Test for Interaction *P*
CIN70	0.82	0.57-1.17	0.82	0.59-1.14	0.99	0.977
CIN25	0.87	0.61-1.29	0.76	0.56-1.05	0.86	0.549
CIN25 in grade III	0.91	0.55-1.48	0.70	0.41-1.00	0.78	0.413
CIN25 in grade I & II	0.74	0.41-1.36	1.27	0.58-2.80	1.76	0.266

In univariate analysis, patients whose tumours had high CIN25 gene expression scores had an increased DRFS (HR: 0.74, 95%CI 0.54-0.99, *p* = 0.046) when treated with E-CMF compared with patients treated with CMF alone (Table 1); a similar association was seen with respect to OS, although this did not reach statistical significance (HR: 0.76, 95%CI 0.56-1.05, *p* = 0.095). Conversely, there was no apparent differential benefit of E-CMF *vs.* CMF in patients with low CIN25 scores for DRFS (HR: 0.85, 95%CI 0.61-1.21, *p* = 0.374) or OS (HR: 0.87, 95%CI 0.61-1.29, *p* = 0.535). A multivariate analysis with adjustment for size, nodal status, ER, pathological grade, HER2, CIN25, treatment and CIN25 by treatment interaction showed only pathological grade, nodal status, tumour size and CEP17 to be significant predictors of outcome (Table 1).

The hazard ratio for the treatment by marker effect of CIN25 was 0.86 (95% CI 0.54-1.36, *p* = 0.519) for DRFS and 0.86 (95% CI 0.53-1.40, *p* = 0.549) for OS (Table 1).

### CIN signature as a biological marker for anthracycline therapy in grade III patients

**Figure 3:**
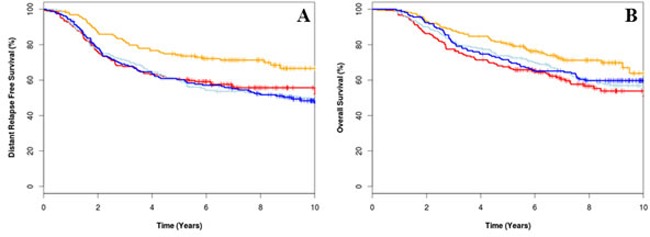
Kaplan-Meier survival curves for epirubicin plus cyclophosphamide, methotrexate and fluorouracil (E-CMF) treated low CIN25 (orange), E-CMF high CIN25 (red), CMF treated low CIN25 (light blue), and CMF high CIN25 (dark blue) for distant relapse free survival (A) overall survival (B)

Previous research identified a significant association between CIN gene expression and grade III tumours [9]; therefore, an exploratory analysis was performed in patients with grade III tumours. In univariate analysis, patients with grade III tumours that had high CIN25 gene expression scores had longer DRFS (HR: 0.66, 96%CI 0.46-0.94, *p* = 0.021) and OS (HR: 0.70, 95%CI 0.49-1.00, *p* = 0.05) when treated with E-CMF than those treated with CMF alone (Figure 3, Table 1). By contrast, no significant benefit from E-CMF treatment versus CMF treatment was demonstrated in patients whose tumours were grade 3 and had low CIN25 gene expression (Table 1). No significant benefit from adjuvant E-CMF versus CMF was demonstrated in patients with grade I and II tumours irrespective of CIN25 gene expression scores (Table 1).

The uncorrected hazard ratio for treatment by marker effect of CIN25 in grade III tumours was 0.81 (95% CI 0.45-1.46, *p* = 0.479) for DRFS and 0.78 (95% CI 0.42-1.43, *p* = 0.413) for OS (Table 1).

### CIN4 as a predictor for anthracycline benefit

In order to select a minimal set of genes that reflects CIN we used the merged clinical cohort (containing both BR9601 and MA.5) and applied a bootstrap aggregation approach to allow a training and validation approach to be tested (see methods).

**Figure 4:**
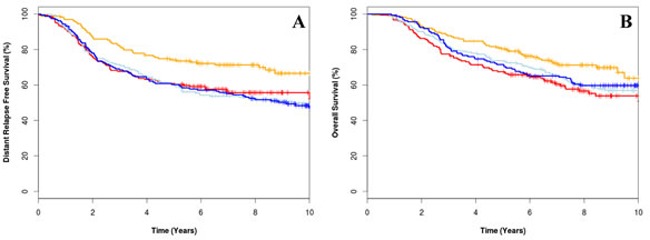
Kaplan-Meier survival curve for low CIN4 score treated with epirubicin plus cyclophosphamide, methotrexate and fluorouracil (E-CMF) (orange line), high CIN4 score treated with E-CMF (red line), low CIN4 score treated with CMF (light blue line) and high CIN4 score treated with CMF (dark blue line) for distant relapse free survival for overall survival (A) and distant relapse free survival (B)

A CIN4 score was generated using the expression values of 4 genes, HDGF, KIAA0286, RFC4 and MSH6, weighted by their regression coefficients. Patients with cancers that have a low CIN4 score had superior DRFS (HR 2.72, 95%CI 1.48-5.02, *p* = 0.001) and OS (HR 2.00, 95%CI 1.09-3.69, *p* = 0.03) when treated with the anthracycline compared to CMF alone (Figure 4). In multivariate analysis, the hazard ratio for treatment marker effect of CIN4 was 0.35 (95% CI 0.15-0.79, *p* = 0.01) for DRFS and 0.35 (95%CI 0.15-0.80, *p* = 0.01) (Table 2).

**Table 2:** Multivariate analysis for DRFS and OS

	Hazard ratio	95% CI	P value
**DRFS**			
CIN4 (continuous score)	8.14	2.18-30.38	0.001
HER2 status	1.32	0.98-1.77	0.06
PgR status	1.15	0.77-1.71	0.50
Tumour Size	1.76	1.29-2.40	0.003
Tumour grade	1.66	1.23-2.84	<0.0001
Nodal status	2.24	1.77-2.84	<0.0001
Treatment	0.60	0.30-1.18	0.14
CIN4 by treatment interaction	0.35	0.15-0.79	0.01
**OS**			
CIN4 (continuous score)	5.84	1.57-21.77	0.008
HER2 status	1.29	0.95-1.75	0.09
PgR status	1.19	0.78-1.81	0.41
Tumour Size	1.79	1.28-2.51	0.0006
Tumour grade	1.97	1.41-2.75	<0.0001
Nodal status	2.17	1.69-2.78	<0.0001
Treatment	0.56	0.28-1.15	0.11
CIN4 by treatment interaction	0.35	0.15-0.80	0.01

## DISCUSSION

Chromosome segregation is a tightly orchestrated process and when this process goes awry CIN and aneuploidy results [16]. The original CIN signature produced by Carter and colleagues used aneuploidy as a surrogate marker for CIN and both this and mRNA CIN signatures were associated with poor prognosis in multiple cancers [11–12]. Furthermore, a study performed by Szasz and colleagues identified a four gene signature, AURKA, FOXM1, TOP2A and TPX2 from the CIN70 signature based on the high level of correlation with histological grade and *in silico* expression [17]. Their four gene score was able to stratify grade 2 breast cancer patients into good and poor prognostics cohorts even better than Ki67 and the mitotic index [17]. CIN has also been linked to sensitivity to specific chemotherapy agents, including anthracyclines [10]. In this study we examined the role of CIN gene signatures as predictive biomarkers for anthracycline benefit in breast cancer.

In a prospectively planned molecular analysis of two retrospective anthracycline trials univariate analyses and an analysis including stratification by grade, data for the CIN25 signature further supported our hypothesis that CIN is associated with anthracycline sensitivity in early breast cancer. However we were unable to demonstrate statistically significant predictive value for the predefined CIN25 and CIN70 signatures in multivariate regression analyses, when correcting for conventional prognostic markers and including the previously validated CEP17 predictive biomarker [10–7]. Several studies have demonstrated correlations between grade and chromosomal instability [10–18–19]. Moreover, low and intermediate grade tumours have fewer structural genomic aberrations and numerical aberrations in whole chromosomes [18–20], perhaps reflecting a reduced incidence of CIN. This may explain why, in our study there appeared to be benefit from the addition of an anthracycline in patients with high grade tumours and high CIN25 signature but not those with grade 1 and 2 tumours. To validate the observed effect of CIN25 as a predictor of anthracycline sensitivity in an independent dataset with 80% power at the *p* = 0.05 level we would require a 2470 patients.

We also used a training and validation approach to derive a novel, minimum 4 gene signature “CIN4” (US patent number 62/024,729) as a predictive biomarker for anthracycline benefit. Using genes within the CIN70 gene panel and a combination of expression clustering, bootstrap aggregation and Cox regression modelling, we were able to identify a set of 4 genes, HDGF, KIAA0286, RFC4 and MSH6 which were significantly predictive of anthracycline benefit. Our study demonstrated that high CIN4 score was associated with improved DRFS in patients treated with anthracycline therapy.

There are a number of multigene signatures associated with prognosis available commercially [21], some of which are being tested in prospective randomized trials such as “Microarray in Node-Negative Disease May Avoid Chemotherapy Trial” (MINDACT) [22] and “Trial Assigning Individualised Options for Treatment (Rx)” (TAILORx) [23]; both studies are assessing whether it is possible to avoid the use of chemotherapy in patients predicted not to benefit. However, no signatures that are specifically associated with benefit from specific chemotherapy agents have been validated to level I evidence as described by Simon et al [24]. At this point it still remains largely unclear which subpopulation of patients will benefit from adjuvant anthracycline therapy and which patients are being treated unnecessarily. Therefore a validated diagnostic test is urgently required to identify which subgroup of patients should be treated with anthracycline and which should be offered alternative therapies. The CIN4 signature identified in this study is a credible candidate to address this specific challenge.

Our CIN4 gene signature differs from existing candidates in being an independent predictor of anthracycline benefit. Interestingly, three of the four genes in our CIN4 signature are involved in DNA repair/DNA-binding activity. Anthracyclines are thought to exert their actions by intercalation with DNA, generation of free radicals and crosslinking DNA to proteins [25]. Therefore, dysregulation of genes involved in DNA repair may plausibly lead to anthracycline sensitivity. To validate this gene signature in an independent dataset with 80% power at the *p* = 0.05 level would require only 300 patients treated in an adjuvant anthracycline trial. In conclusion we have demonstrated that a 4 gene signature, related to CIN, represents an independent predictor of anthracycline sensitivity.

## MATERIALS AND METHODS

### Patients

#### BR9601 trial

The BR9601 trial recruited 374 pre- and post-menopausal women with completely excised, histologically confirmed breast cancer and a clear indication for adjuvant chemotherapy. Patients were randomized between 8 cycles of CMF (i.v. cyclophosphamide 750 mg/m^2^, methotrexate 50 mg/m^2^ and 5-fluorouracil 600 mg/m^2^) every 21 days, and E-CMF (4 cycles of epirubicin 100 mg/m^2^ every 21 days followed by 4 cycles of the same CMF regimen ); patient characteristics are shown in supplementary data (Figure 1, Supplementary Table 1). The protocol was approved by central and local ethics committees, and each patient provided written informed consent prior to randomization. For the current analysis, tissue blocks were retrieved and RNA was extracted. The primary outcomes of the BR9601 study were RFS and OS, however distant relapse free survival was also reported.

#### MA.5 trial

The MA.5 trial randomized 716 premenopausal women with node-positive breast cancer to receive either adjuvant CEF or CMF (Figure 1, Supplementary Table 1). The CEF regimen consisted of 6 cycles of epirubicin 60 mg/m^2^ and 5-fluorouracil (5-FU) 500 mg/m^2^, both delivered intravenously on days 1 and 8, and oral cyclophosphamide 75 mg/m^2^ daily on days 1 through 14. Patients randomized to the CEF regimen also received antibiotic prophylaxis throughout. The CMF regimen consisted of 6 cycles of methotrexate 40 mg/m^2^ and 5-FU 600 mg/m^2^, both delivered intravenously on days 1 and 8, and oral cyclophosphamide 100 mg/m^2^ daily on days 1 through 14. The MA.5 protocol was approved by the institutional review board at each participating center and registered as NCI-V90-0027 on cancer.gov. Written informed consent was obtained from each woman.

### RNA extraction

Total RNA from FFPE tissue samples (2 × 10μM full sections) were extracted using the RecoverAll Total Nucleic Acid Isolation kit (Life Technologies) according to the manufacturers protocol and concentrations were determined using the NanoDrop ND-1000 spectrophotometer (NanoDrop Technologies).

### Gene expression analysis

RNA (400ng) was used with the nCounter system, according to the manufacturer's protocol (NanoString ^®^ Technologies, Seattle, WA, USA). In brief, 5μl of RNA was hybridized at 96°C overnight with the NanoString Codeset. Probes for the analysis of test genes and controls were synthesized by NanoString technologies, including probes for the 70 genes of interest and 6 normalising genes (Supplementary Table 2). All 76 genes and controls were assayed simultaneously in multiplexed reactions. After probe hybridizations and NanoString nCounter digital reading, counts for each RNA species were extracted and analyzed. The nCounter CodeSet contains two types of built-in controls: positive controls (spiked RNA at various concentrations to assess the overall assay performance) and negative controls (probes for background calculation). The raw data were normalized to the standard curve generated via the nCounter system spike-in controls present in all reactions.

### Statistics

The SPSS (v20) statistical package was used for statistical analysis. Kaplan-Meier estimates of survival were used for analysis of distant relapse free (DRFS) and overall survival (OS). The Cox's proportional hazard model was used to obtain hazard ratios for relapse or death. When comparing outcomes between the treatment arms within the groups of patients identified by biomarker expression, p-values were not calculated for sub-groups to avoid multiple testing and bias where one group was much smaller than the other. The Cox model was instead used to identify statistically significant interactions (*p* < 0.05) between biomarkers and outcome on the different treatments (treatment by marker effect), in models that also included biomarker status (marker effect) and treatment, as covariates. For the CIN25 and CIN70 signatures values were dichotomised around the median as previously described and a combined, preplanned treatment by marker analysis was performed using results from both the BR9601 and MA5 clinical trial cohorts.

### Development of a 4 gene predictive signature

The combined BR9601/MA5 cohort was split to two groups according to the randomized treatment. Using Affinity propagation clustering (R package apcluster^1^), the 70 genes were clustered into 9 groups according to their expression profiles (Supplementary Figure 2). A multivariate Cox model was fitted for each gene, adjusting for clinical variables including HER2, ER, PgR, tumour size, grade and nodal status. The top genes from each expression cluster, with the most significant p-value in the anthracycline treated cohort and a non-significant CMF cohort, were selected to make a list of 21 genes (Supplementary Figure 1). The number of genes selected from each cluster was weighted by the size of the cluster; as a result, more genes were selected for large clusters compared to small clusters (Supplementary Table 4). Each cluster had to be represented at least once even if the gene in that cluster was not significant. The gene breakdown for clusters 1-9 was as follows: CDC2; KIF20A; HDGF; MDUFAB1, CDC3A; CDC6, MAD2L1, NXT1, TOPK; FEN1, CCT5; DKC1, ECT2; KIAA0286, MCM2, RFC4, MSH6; ch.TOG, CNAP1, TOP2A, RRM1 (Supplementary Table 4). From this list, all possible combinations of 2, 3, 4 and 5 gene signatures were examined (210, 1330, 5985, and 20349 combinations respectively). Using bootstrapped aggregation (bagging), each combination was bootstrapped 100 times, with the median area under the curve (AUC) noted as the bagged variable. In each bootstrap, the treatment cohort was split into 60% training and 40% test sets [26–27–28–29]. The AUC was calculated from the test sets (R package survivalROC^2^) (Supplementary Table 5). The gene signature selected had the greatest AUC for a treatment by marker effect in both the training and validation approaches and included four genes, HDGF, KIAA0286, RFC4 and MSH6, termed the CIN4 signature. A multivariate Cox regression was fitted using the four genes, adjusting for the same clinical variables mentioned above (Supplementary Table 6). A CIN4 score was generated using the expression values of the four genes, weighted by their regression coefficients.

## SUPPLEMENTARY MATERIAL TABLES AND FIGURES



## References

[1] (2005). Effects of chemotherapy and hormonal therapy for early breast cancer on recurrence and 15-year survival: an overview of the randomised trials.

[2] Swain S. M, Whaley F. S, Ewer M. S (2003). Congestive heart failure in patients treated with doxorubicin: a retrospective analysis of three trials. Cancer.

[3] Mercuro G, Cadeddu C, Piras A, Dessi M, Madeddu C, Deidda M, Serpe R, Massa E, Mantovani G (2007). Early epirubicin-induced myocardial dysfunction revealed by serial tissue Doppler echocardiography: correlation with inflammatory and oxidative stress markers. Oncologist.

[4] Jones L. W, Haykowsky M. J, Swartz J. J, Douglas P. S, Mackey J. R (2007). Early breast cancer therapy and cardiovascular injury. J. Am. Coll. Cardiol.

[5] Pritchard K. I, Shepherd L. E, O'Malley F. P, Andrulis I. L, Tu D, Bramwell V. H, Levine M. N (2006). HER2 and responsiveness of breast cancer to adjuvant chemotherapy.

[6] Bartlett J. M, Munro A. F, Dunn J. A, McConkey C, Jordan S, Twelves C. J, Cameron D. A, Thomas J, Campbell F. M, Rea D. W, Provenzano E, Caldas C, Pharoah P, Hiller L, Earl H, Poole C. J (2010). Predictive markers of anthracycline benefit: a prospectively planned analysis of the UK National Epirubicin Adjuvant Trial (NEAT/BR9601). Lancet Oncol.

[7] Pritchard K. I, Munro A, O'Malley F. P, Tu D, Li X, Levine M. N, Shepherd L, Chia S, Bartlett J. M (2012). Chromosome 17 centromere (CEP17) duplication as a predictor of anthracycline response: evidence from the NCIC Clinical Trials Group (NCIC CTG) MA.5 Trial. Breast Cancer Res Treat.

[8] Bartlett J. M. S, Desmedt C, Munro A, O'Malley F. P, Larsimont D, Di Leo A, Cameron D. A, Isola J, Shepherd L, Twelves C. J, Pritchard K. I, TIIa, Metaanal Grp (2009). Chromosome 17 polysomy: a unifying hypothesis underlying benefit from adjuvant anthracyclines?. Cancer Research.

[9] Bartlett J. M, McConkey C. C, Munro A. F, Desmedt C, Dunn J. A, Larsimont D. P, O'Malley F. P, Cameron D. A, Earl H. M, Poole C. J, Shepherd L. E, Cardoso F, Jensen M. B, Caldas C, Twelves C. J, Rea D. W, Ejlertsen B, Di Leo A, Pritchard K. I (2015). Predicting Anthracycline Benefit: TOP2A and CEP17-Not Only but Also. J. Clin. Oncol.

[10] Munro A. F, Twelves C, Thomas J. S, Cameron D. A, Bartlett J. M (2012). Chromosome instability and benefit from adjuvant anthracyclines in breast cancer. Br J Cancer.

[11] Carter S. L, Eklund A. C, Kohane I. S, Harris L. N, Szallasi Z. A (2006). signature of chromosomal instability inferred from gene expression profiles predicts clinical outcome in multiple human cancers. Nat Genet.

[12] Habermann J. K, Doering J, Hautaniemi S, Roblick U. J, Bundgen N. K, Nicorici D, Kronenwett U, Rathnagiriswaran S, Mettu R. K, Ma Y, Kruger S, Bruch H. P, Auer G, Guo N. L, Ried T (2009). The gene expression signature of genomic instability in breast cancer is an independent predictor of clinical outcome. Int J Cancer.

[13] Munro A. F, Cameron D. A, Bartlett J. M (2010). Targeting anthracyclines in early breast cancer: new candidate predictive biomarkers emerge. Oncogene.

[14] Carter S. L, Eklund A. C, Kohane I. S, Harris L. N, Szallasi Z. A (2006). signature of chromosomal instability inferred from gene expression profiles predicts clinical outcome in multiple human cancers. Nat Genet.

[15] Swanton C, Nicke B, Schuett M, Eklund A. C, Ng C, Li Q, Hardcastle T, Lee A, Roy R, East P, Kschischo M, Endesfelder D, Wylie P, Kim S. N, Chen J. G, Howell M, Ried T, Habermann J. K, Auer G, Brenton J. D, Szallasi Z, Downward J (2009). Chromosomal instability determines taxane response. Proc Natl Acad Sci U S A.

[16] Bakhoum S. F, Compton D. A (2012). Chromosomal instability and cancer: a complex relationship with therapeutic potential. J Clin Invest.

[17] Szasz A. M, Li Q, Eklund A. C, Sztupinszki Z, Rowan A, Tokes A. M, Szekely B, Kiss A, Szendroi M, Gyorffy B, Szallasi Z, Swanton C, Kulka J (2013). The CIN4 chromosomal instability qPCR classifier defines tumor aneuploidy and stratifies outcome in grade 2 breast cancer. PLoS One.

[18] A'Hern R. P, Jamal-Hanjani M, Szasz A. M, Johnston S. R, Reis-Filho J. S, Roylance R, Swanton C (2013). Taxane benefit in breast cancer—a role for grade and chromosomal stability. Nat Rev Clin Oncol.

[19] Sauer T, Beraki K, Jebsen P. W, Ormerod E, Naess O (1997). Ploidy analysis by in situ hybridization of interphase cell nuclei in fine-needle aspirates from breast carcinomas: correlation with cytologic grading. Diagn. Cytopathol.

[20] Dellas A, Torhorst J, Schultheiss E, Mihatsch M. J, Moch H (2002). DNA sequence losses on chromosomes 11p and 18q are associated with clinical outcome in lymph nodenegative ductal breast cancer. Clin Cancer Res.

[21] Harbeck N, Sotlar K, Wuerstlein R, Doisneau- Sixou S (2014). Molecular and protein markers for clinical decision making in breast cancer: today and tomorrow. Cancer Treat Rev.

[22] Rutgers E, Piccart-Gebhart M. J, Bogaerts J, Delaloge S, Veer L. V, Rubio I. T, Viale G, Thompson A. M, Passalacqua R, Nitz U, Vindevoghel A, Pierga J. Y, Ravdin P. M, Werutsky G, Cardoso F (2011). The EORTC 10041/BIG 03-04 MINDACT trial is feasible: results of the pilot phase. Eur J Cancer.

[23] Zujewski J. A, Kamin L (2008). Trial assessing individualized options for treatment for breast cancer: the TAILORx trial. Future. Oncol.

[24] Simon R. M, Paik S, Hayes D. F (2009). Use of archived specimens in evaluation of prognostic and predictive biomarkers. J Natl Cancer Inst.

[25] Minotti G, Recalcati S, Menna P, Salvatorelli E, Corna G, Cairo G (2004). Doxorubicin cardiotoxicity and the control of iron metabolism: quinone-dependent and independent mechanisms. Methods Enzymol.

[26] Govindan G, Nair A. S (2013). Bagging with CTD—a novel signature for the hierarchical prediction of secreted protein trafficking in eukaryotes. Genomics Proteomics Bioinformatics.

[27] Tan A. C, Gilbert D (2003). Ensemble machine learning on gene expression data for cancer classification. Appl. Bioinformatics.

[28] Zhang H, Singer BH (2010). Recursive partitioning and applications.

[29] Breiman L (1996). Bagging Predictors. Machine Learning.

