# Supplementary material for: A four gene signature predicts benefit from anthracyclines: evidence from the BR9601 and MA.5 clinical trials
Source: Oncotarget. 2015 Sep 10;6(31):31693–701. doi: 10.18632/oncotarget.5562 (PMC4741633; doi:10.18632/oncotarget.5562)
Supplement: Supplementary file 1 [file oncotarget-06-31693-s001.pdf]

A four gene signature predicts benefit from anthracyclines: evidence from the BR9601 and MA.5 clinical trials

Supplementary Material

Supplementary Table 1: Experimental and housekeeping genes used in the gene expression study.

	Clinical trial		CIN analysis	
	BR9601	MA.5	BR9601	MA.5
Number	374	710	282	421
Age<br>Mean (range)	50.6<br>(22.7-76.0)	43.9<br>(23.4-57.2)	50.6<br>(26.2-76.0)	43.9<br>(23.4-57.2)
Treatment<br>E-CMF<br>CMF	183 (48.9%)<br>191 (51.1%)	350 (49.3%)<br>360 (50.7%)	138 (48.9)<br>144 (51.1%)	208 (49.4%)<br>213 (50.6%)
Size<br><2.0 cm<br>>2.0 cm<br>Missing	123 (32.9%)<br>251 (67.1%)	265 (37.9%)<br>435 (62.1%)<br>10	94 (33.3%)<br>188 (66.7)	150 (36.1%)<br>265 (63.9%)
Nodes<br>0<br>1-3<br>≥4	48 (12.8%)<br>214 (57.3%)<br>112 (29.9%)	0<br>433 (61.0%)<br>277 (39.0%)		
Grade<br>I<br>II<br>III<br>Unknown	22 (6.1%)<br>126 (35.2%)<br>210 (58.7%)<br>16	77 (12.4%)<br>204 (32.9%)<br>340 (54.8%)<br>89	19 (6.8%)<br>96 (34.3%)<br>165 (58.9%)<br>2	42 (10.2%)<br>120 (29.1%)<br>250 (60.7%)<br>9
ER Status<br>Positive<br>Negative<br>Unknown	202 (62.9%)<br>119 (37.1%)<br>53	424 (59.7%)<br>200 (32.1%)<br>86	155 (62.8%)<br>92 (37.2%)<br>35	253 (67.3%)<br>123 (32.7%)<br>45

Supplementary Table 2: Patient and tumour characteristics from the BR9601 and MA.5 study.

Experimental genes						Housekeeping genes
DHCR7	CCNB2	ch-TOG	RNASEH2A	AURKB	ATM	GUSB
GPIandMGC13096	FEN1	MSH6	RRM1	CDC2	ATR	PUM1
CKS2	FLJ10036	PCNA	RRM2	MAD2L1	CDC25B	SF3A1
BRRN1	H2AFX	RAD21	TGIF2	PRC1	CDC25C	TBP
CNAP1	H2AFZ	RFC4	ATAD2	TPX2	CHEK1	TFRC
MCM10	HDGF	UNG	NDUFAB1	TTK	CHEK2	TMED10
CDC20	KIF4A	CDC45L	KIAA0286	UBE2C	MDM2	
ESPL1	PTTG1	CDC6	KIF20A	ZWINT	P53	
FOXM1	AURKA	CDCA8	CDC3A	CMAS	CDKN1A	
MTB	MELK	CEP55	ACTL6A	DKC1		
NEK2	RAD51AP1	CTPS	LSM4	TRIP13		
OIP5	TOPK	ECT2	SFRS2	CCT5		
TOP2A	EZH2	MCM2	ELAV1	MTCH2		
CCNB1	ASF1B	MCM7	NXT1	NUP205		

Supplementary Table 3: Cox regression p-values for each of the 70 genes adjusted for age, her2 status, tumour size, nodal status and grade. The test is reported for CMF and E-CMF separately.

GENE	**E-CMF (Logrank p-value)**	**CMF (Logrank p-value)**	**abs_diff**
ACTL6A	0.1606349758	0.1969687045	0.0363337288
ASF1B	0.4117646207	0.703358929	0.2915943083
ATAD2	0.488406011	0.6554240724	0.1670180614
AURKA	0.6602990844	0.6923896068	0.0320905224
AURKB	0.4307066035	0.4445707696	0.0138641661
BRRN1	0.3335476259	0.449727395	0.1161797691
CCNB2	0.3360263818	0.6466923032	0.3106659214
CCT5	0.1448548819	0.6590259471	0.5141710652
CDC2	0.6658085123	0.4941615767	0.1716469356
CDC20	0.6252491199	0.6512055177	0.0259563978
CDC3A	0.315004798	0.5547353695	0.2397305715
CDC45L	0.5470779698	0.5479149184	0.0008369486
CDC6	0.0141017227	0.6995651074	0.6854633847
CDCA8	0.6261004313	0.4317983512	0.1943020801
CEP55	0.5590902344	0.6252462698	0.0661560355
ch.TOG	0.1988997278	0.5798667664	0.3809670386
CKS2	0.3771051754	0.5702507861	0.1931456107
CMAS	0.5927651204	0.3273865094	0.2653786111
CNAP1	0.0984148803	0.600454591	0.5020397107
CTPS	0.5757974593	0.6490918956	0.0732944362
DHCR7	0.6199837409	0.6504258335	0.0304420927
DKC1	0.2057812012	0.6858676887	0.4800864875
ECT2	0.0531218024	0.3567372791	0.3036154768
ELAV1	0.6854236049	0.5329087012	0.1525149037
ESPL1	0.6378507026	0.5263607538	0.1114899488
EZH2	0.464457859	0.6035971115	0.1391392525
FEN1	0.0445992969	0.5687032396	0.5241039427
FLJ10036	0.6394147546	0.5882767156	0.051138039
FOXM1	0.5400643501	0.3129773393	0.2270870108
GPIandMGC13096	0.6642023789	0.5786215227	0.0855808562
H2AFX	0.5582253342	0.2488925091	0.3093328252
H2AFZ	0.4622153605	0.5936526706	0.1314373101
HDGF	0.5814040026	0.7130194707	0.1316154681
KIAA0286	0.1247435823	0.6244212204	0.4996776381
KIF20A	0.6914555924	0.6099811681	0.0814744244
KIF4A	0.1644159396	0.5967235245	0.4323075849
LSM4	0.2067163078	0.4782823703	0.2715660626
MAD2L1	0.0625004801	0.6873002296	0.6247997495
MCM10	0.6338287685	0.4138837343	0.2199450343
MCM2	0.1225851629	0.6301826229	0.50759746
MCM7	0.6080002173	0.5852090958	0.0227911215
MELK	0.6986319883	0.6396273131	0.0590046751
MSH6	0.0526608675	0.6868566448	0.6341957773
MTB	0.3247441368	0.500331132	0.1755869951
MTCH2	0.6006042944	0.6893977834	0.088793489
NDUFAB1	0.5886245391	0.2576425768	0.3309819623

NEK2	0.3577263665	0.6067211331	0.2489947666
NUP205	0.2540036122	0.5916351913	0.3376315791
NXT1	0.1493520782	0.638278526	0.4889264477
OIP5	0.6947498905	0.4769069304	0.2178429601
P53	0.2441970634	0.3207519232	0.0765548598
PCNA	0.4238261725	0.3914174447	0.0324087278
PRC1	0.3249420857	0.649935443	0.3249933573
PTTG1	0.3822107911	0.6072511063	0.2250403152
RAD21	0.6274389485	0.6624756278	0.0350366793
RAD51AP1	0.6957520964	0.4291592885	0.2665928079
RFC4	0.0484459568	0.5787751713	0.5303292145
RNASEH2A	0.49300782	0.6139205205	0.1209127005
RRM1	0.1268867673	0.6647919672	0.5379051998
RRM2	0.4532817481	0.5717334261	0.118451678
SFRS2	0.6193218823	0.5007884886	0.1185333937
TGIF2	0.3035038317	0.604282069	0.3007782373
TOP2A	0.1524154757	0.6627474543	0.5103319786
TOPK	0.2054906515	0.5975762262	0.3920855747
TPX2	0.5936673384	0.6122924581	0.0186251197
TRIP13	0.1466730217	0.5661660081	0.4194929863
TTK	0.4867482167	0.5597341007	0.072985884
UBE2C	0.509325873	0.6631031477	0.1537772747
UNG	0.7203736204	0.6751633809	0.0452102395
ZWINT	0.109906396	0.2334294449	0.1235230488

Supplementary Table 4: Cluster breakdown of the 21 genes selected to be considered for the multi-gene signature development.

CLUSTER	CLUSTER SIZE	Number of selected genes	Genes
1	3	1	CDC2
2	1	1	KIF20A
3	1	1	HDGF
4	6	2	NDUFAB1, CDC3A
5	15	4	CDC6, MAD2L1, NXT1, TPOK
6	7	2	FEN1, CCT5
7	8	2	DKC1, ECT2
8	15	4	KIAA0286, MCM2, RFC4, MSH6
9	14	4	Ch.TOG, CNAP1, TOP2A, RRM1

Supplementary Table 5: Top 10 AUC results for the 2,3,4, and 5 gene models. AUCs are reported for CMF and E-CMF along with the delta AUC.

Genes	E-CMF Median AUC	CMF Median AUC	Number of genes
**CDC2:KIAA0286***	**0.573038029**	**0.499944028**	**2**
HDGF:KIAA0286	0.569445819	0.470480025	2
CDC2:NXT1	0.565964926	0.572359704	2
NDUFAB1:KIAA0286	0.564186305	0.489798048	2
HDGF:MSH6	0.563159097	0.481925435	2
DKC1:KIAA0286	0.562687355	0.487621351	2
NXT1:ch.TOG	0.560987486	0.517239312	2
KIF20A:NXT1	0.560486257	0.55231784	2
CDC3A:NXT1	0.560332464	0.54901104	2
HDGF:RRM1	0.559458075	0.50775364	2
**HDGF:KIAA0286:ch.TOG***	**0.58033007**	**0.465561863**	**3**
CDC2:KIAA0286:RFC4	0.578075141	0.496638028	3
HDGF:RFC4:MSH6	0.577670337	0.484792168	3
CDC2:HDGF:KIAA0286	0.577641232	0.480324824	3
HDGF:NXT1:ch.TOG	0.57701026	0.502151561	3
CDC2:NXT1:KIAA0286	0.571277779	0.548064157	3
CDC2:NXT1:RRM1	0.570177615	0.559652671	3
HDGF:DKC1:RRM1	0.569450045	0.517086305	3
CDC2:KIAA0286:ch.TOG	0.56828609	0.498828367	3
NXT1:KIAA0286:RFC4	0.564617594	0.494655011	3
**HDGF:KIAA0286:RFC4:MSH6***	**0.581076029**	**0.478407495**	**4**
HDGF:CCT5:RFC4:MSH6	0.579835252	0.476213941	4
CDC2:HDGF:NXT1:KIAA0286	0.576588268	0.518225798	4
CDC2:HDGF:KIAA0286:RFC4	0.576272997	0.488857334	4
CDC2:HDGF:KIAA0286:RRM1	0.570256164	0.499709829	4
CDC2:HDGF:CCT5:RFC4	0.569930223	0.501191458	4
HDGF:DKC1:KIAA0286:MSH6	0.569747609	0.484703296	4
KIF20A:HDGF:KIAA0286:RRM1	0.569685185	0.486300361	4
HDGF:CCT5:KIAA0286:RFC4	0.568346336	0.476167046	4
HDGF:RFC4:MSH6:RRM1	0.567643717	0.482650289	4
**CDC2:HDGF:KIAA0286:RFC4:RRM1***	**0.578693166**	**0.495601865**	**5**
HDGF:NXT1:KIAA0286:RFC4:MSH6	0.573915377	0.485380106	5
CDC2:HDGF:NXT1:RFC4:MSH6	0.572193653	0.502609797	5
CDC2:HDGF:KIAA0286:RFC4:MSH6	0.570832698	0.493234599	5
CDC2:HDGF:CCT5:KIAA0286:RFC4	0.5702546	0.494509483	5
CDC2:KIF20A:HDGF:KIAA0286:RFC4	0.569788391	0.489827349	5
CDC2:HDGF:KIAA0286:MCM2:RFC4	0.568764021	0.484211458	5
KIF20A:HDGF:KIAA0286:RFC4:MSH6	0.567573989	0.472420735	5
CDC2:HDGF:NXT1:KIAA0286:RFC4	0.567335041	0.49541865	5
CDC2:HDGF:KIAA0286:RFC4:ch.TOG	0.565731164	0.481541752	5

Supplementary Table 6: Hazard ratios for the CIN4 signature, adjusted for clinical variables a) using distance relapse free survival (DRFS) and b) Overall Survival (OS).

A

Distant relapse Free Survival (DRFS)							
	Treatment by Marker Interaction						
	**coef**	**exp(coef)**	**se(coef)**	**z**	**Pr(>|z|)**	**Lower 95% CI**	**Upper 95% CI**
CIN4 (continuous score)	2.0976	8.1465	0.6716	3.123	0.001789	2.1842	30.3837
HER2	0.2767	1.3187	0.1491	1.856	0.063484	0.9846	1.7663
PgR	0.1383	1.1483	0.2042	0.677	0.498185	0.7696	1.7135
Size (binned)	0.5656	1.7604	0.1583	3.572	0.000354	1.2908	2.401
Path Grade(binned)	0.5096	1.6647	0.1537	3.316	0.000915	1.2317	2.2499
NODE	0.8069	2.2409	0.1214	6.645	3.04E-011	1.7663	2.843
Treatment	-0.5116	0.5995	0.3455	-1.481	0.138704	0.3046	1.1801
CIN4*Treat Interaction	-1.056	0.3478	0.4154	-2.542	0.011015	0.1541	0.7852
*stratified by ER							
E-CMF Treatment							
	**coef**	**exp(coef)**	**se(coef)**	**z**	**Pr(>|z|)**	**Lower 95% CI**	**Upper 95% CI**
CIN4 (continuous score)	1.0016	2.7226	0.3122	3.209	0.00133	1.4767	5.02
HER2	0.2491	1.2828	0.2413	1.032	0.30191	0.7995	2.058
PgR	-0.2489	0.7796	0.3434	-0.725	0.46847	0.3978	1.528
Size (binned)	0.5887	1.8016	0.2309	2.549	0.0108	1.1458	2.833
Path Grade(binned)	0.3196	1.3766	0.2273	1.406	0.15975	0.8817	2.149
NODE	0.7628	2.1442	0.1864	4.092	4.27E-005	1.488	3.09
Treatment	1.0016	2.7226	0.3122	3.209	0.00133	1.4767	5.02
CIN4*Treat Interaction	0.2491	1.2828	0.2413	1.032	0.30191	0.7995	2.058
*stratified by ER							
CMF Treatment							
	**coef**	**exp(coef)**	**se(coef)**	**z**	**Pr(>|z|)**	**Lower 95% CI**	**Upper 95% CI**
CIN4 (continuous score)	0.02636	1.02671	0.29412	0.09	0.92859	0.5769	1.827
HER2	0.30044	1.35046	0.19484	1.542	0.12308	0.9218	1.978
PgR	0.36944	1.44692	0.25514	1.448	0.14763	0.8775	2.386
Size (binned)	0.56123	1.75283	0.21831	2.571	0.01015	1.1427	2.689
Path Grade(binned)	0.64563	1.90719	0.21105	3.059	0.00222	1.2611	2.884
NODE	0.82767	2.28799	0.16224	5.102	3.37E-007	1.6648	3.144
Treatment	0.02636	1.02671	0.29412	0.09	0.92859	0.5769	1.827
CIN4*Treat	0.30044	1.35046	0.19484	1.542	0.12308	0.9218	1.978

Interaction							
*stratified by ER	0.36944	1.44692	0.25514	1.448	0.14763	0.8775	2.386

B

Overall Survival (DRFS)							
	**Treatment by Marker Interaction**						
	**coef**	**exp(coef)**	**se(coef)**	**z**	**Pr(>|z|)**	**Lower 95% CI**	**Upper 95% CI**
CIN4 (continuous score)	0.694362	2.002431	0.31164	2.228	0.025875	1.0871	3.688
HER2	0.099964	1.105131	0.2517	0.397	0.69125	0.6748	1.81
PgR	-0.00572	0.994301	0.36838	-0.016	0.987621	0.483	2.047
Size (binned)	0.48639	1.626434	0.23795	2.044	0.040948	1.0202	2.593
Path Grade(binned)	0.557098	1.745599	0.2477	2.249	0.024508	1.0742	2.837
NODE	0.693257	2.00022	0.19323	3.588	0.000334	1.3696	2.921
**E-CMF Treatment**							
	**coef**	**exp(coef)**	**se(coef)**	**z**	**Pr(>|z|)**	**Lower 95% CI**	**Upper 95% CI**
CIN4 (continuous score)	-0.3043	0.7377	0.3065	-0.993	0.3209	0.4045	1.345
HER2	0.3929	1.4812	0.2038	1.927	0.0539	0.9934	2.209
PgR	0.29	1.3364	0.2659	1.09	0.2755	0.7936	2.25
Size (binned)	0.734	2.0834	0.2513	2.92	0.0035	1.273	3.41
Path Grade(binned)	0.7602	2.1387	0.238	3.194	0.0014	1.3415	3.41
NODE	0.8014	2.2286	0.1726	4.642	3.44E-006	1.5889	3.126
**CMF Treatment**							
	**coef**	**exp(coef)**	**se(coef)**	**z**	**Pr(>|z|)**	**Lower 95% CI**	**Upper 95% CI**
CIN4 (continuous score)	1.7656	5.8452	0.6709	2.632	0.008497	1.5693	21.772
HER2	0.2558	1.2915	0.1545	1.656	0.097773	0.9541	1.748
PgR	0.1755	1.1918	0.2143	0.819	0.412749	0.7831	1.814
Size (binned)	0.5825	1.7904	0.1718	3.391	0.000698	1.2786	2.507
Path Grade(binned)	0.6766	1.9672	0.1704	3.971	7.15E-005	1.4087	2.747
NODE	0.7735	2.1673	0.1278	6.05	1.45E-009	1.6869	2.784
Treatment	-0.5773	0.5614	0.3642	-1.585	0.112895	0.275	1.146
CIN4*Treat Interaction	-1.0473	0.3509	0.4211	-2.487	0.012886	0.1537	0.801

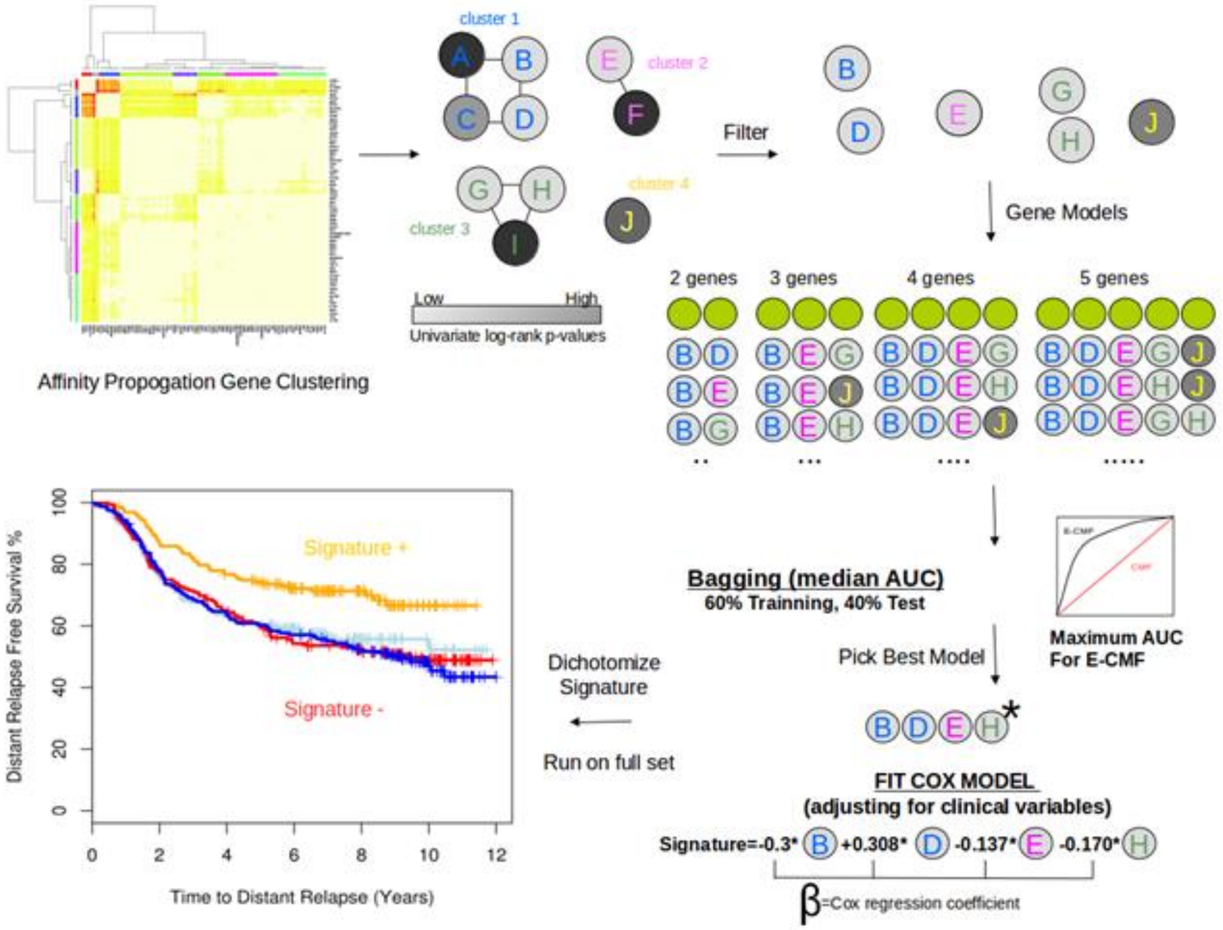

Supplementary Figure 1: Signature Development Overview. Affinity propagation is first used to cluster expression values of the 70 genes. Cox regression test is conducted per gene and the top significant genes from each cluster are selected. All possible combinations of 2,3,4, and 5 genes models are tested. The model with the highest AUC in E-CMF and largest AUC difference between CMF and E-CMF is picked. Signature score is generated by combining the coefficients from the cox model fitted with the selected genes and adjusted for clinical variables.

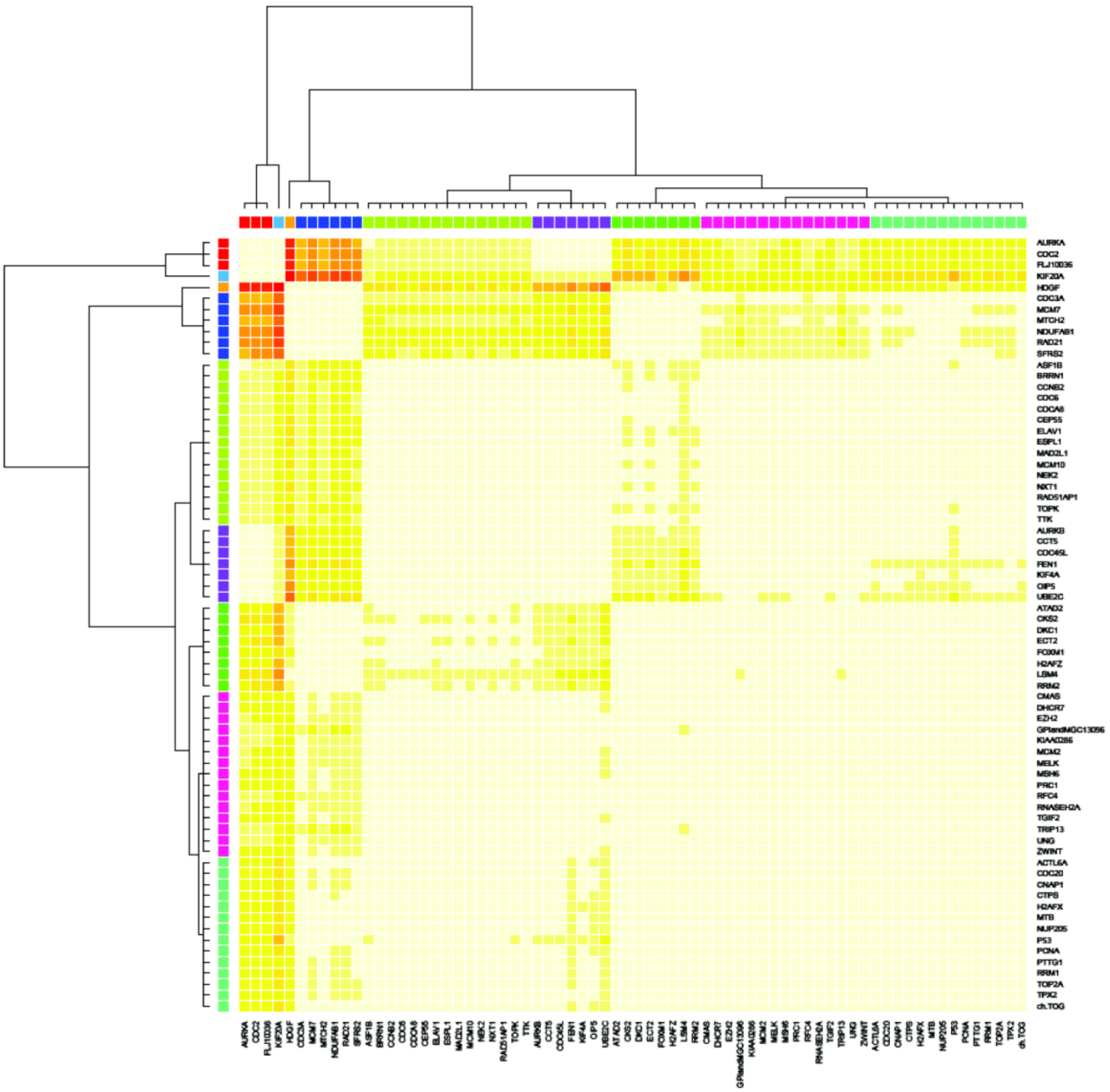

Supplementary Figure 2: Heatmap displaying the results of the affinity propogation expression clustering of the 70 genes.
